# Analysis of characteristics of four patients with adrenal unicentric Castleman disease

**DOI:** 10.3389/fendo.2023.1181929

**Published:** 2023-05-17

**Authors:** Hao Yu, Yuepeng Wang, Yijun Li, Jin Du, Qinghua Guo, Weijun Gu, Zhaohui Lyu, Jingtao Dou, Yiming Mu, Li Zang

**Affiliations:** ^1^ Department of Endocrinology, The First Medical Center of Chinese PLA General Hospital, Beijing, China; ^2^ Department of Endocrinology, General Hospital of Northern Theater Command, Shenyang, China; ^3^ School of Medicine, Nankai University, Tianjin, China

**Keywords:** castleman disease, adrenal gland, lymphoma, adrenal incidentaloma, pheochromocytoma

## Abstract

**Background:**

Castleman Disease (CD) is a group of diseases with characteristic lymph node histopathology, characterized by marked enlargement of deep or superficial lymph nodes. Adrenal CD is rarely reported, and an accurate preoperative diagnosis of adrenal CD is difficult.

**Method:**

We report four cases of CD in the adrenal gland confirmed by pathology and review the characteristics of this rare disease, highlighting the necessity of diagnostic evaluation and follow-up of the patients.

**Results:**

All of the patients sought medical advice because of adrenal incidentalomas. No significant abnormalities were presented in the biochemistry or endocrine systems. The imaging suggested a moderate-to-large mass with uneven moderate contrast enhancement of the adrenal region, similar to a pheochromocytoma. All cases were misdiagnosed as pheochromocytomas before operation and finally confirmed by histopathology. Three cases were pathologically diagnosed as hyaline vascular CD, and one case was diagnosed as plasma cell CD. All the patients are alive without recurrence after a median follow-up of 8 years.

**Conclusion:**

The adrenal CD should be considered after excluding pheochromocytoma and malignancy in the adrenal region. The long-term prognosis of patients with complete resection of the mass is excellent.

## Introduction

Castleman Disease (CD), also named Giant Lymphnode Hyperplasia, is a rare lymphoproliferative disease that can divide into hyaline vascular (HV), plasma cell (PC), and mixed variants on the basis of histological classification. The etiology of CD is still unclear and is associated with the monoclonal proliferation process (1) and increased interleukin-6 (IL-6) (2). A recent study revealed no clear associations between UCD and the activity of HHV-8 or HIV infection (3). The CD was divided into unicentric CD (UCD) and multicentric CD (MCD) according to single or multiple lymph node involvement (4, 5). It can involve all lymph nodes, typically in the mediastinum, cervical, retroperitoneal, and axillary areas, and around 4% of UCD occurs in the adrenal region (6–8). The clinical symptoms of CD are related to the number and location of the affected lymph nodes. The first manifestation is usually the discovery of a slow-growing tumor or systemic symptoms such as fever and weight loss, and the duration of systemic symptoms or lymph node enlargement varies from several weeks to several months. Adrenal UCD is rare and is often misdiagnosed as pheochromocytoma or adrenocortical carcinoma before operation due to its large size and lack of specificity in symptoms and laboratory examinations (9). CD is considered benign in its localized form but aggressive in the multicentric type (10). Because of the variability of clinical presentation, the selection of the appropriate therapeutic approach remains unclear. In the article, we present four cases of adrenal UCD and discuss the clinical manifestations, laboratory results, imaging and pathological features, management, and follow-up of this rare disorder.

## Materials and methods

We performed a retrospective analysis of the clinical, biochemical, imaging, and pathological characteristics of four patients with a single focus on adrenal CD who were managed with surgical resection. Patients were diagnosed at the Chinese PLA General Hospital from September 2008 to February 2022. Each patient underwent surgery, and CD was then confirmed pathologically after surgery. Patients were followed up to supplement outcome information until December 1st, 2022. Our study was approved by the Ethics Committee of the First Medical Center of Chinese PLA General Hospital (approval number: S2021-554-01).

## Results

### Clinical characteristics

Four patients, two females and two males, between 26 and 58 years of age (mean age: 46 years), were diagnosed with adrenal incidentaloma. Two patients were transferred to the Department of Urology for surgery after detailed evaluation in the Department of Endocrinology (cases 1, 2), while two patients attended urology directly for surgery (cases 3, 4). After further consultation, one patient had prodromal symptoms of fever and palpitations (case 3). No one complained of headaches, sweating, abdominal pain, weight loss, or other typical symptoms of secondary hypertension. No patients presented with Cushing appearance. Physical examination revealed a soft abdomen without palpable mass or peripheral lymphadenopathy. One patient was treated with monotherapy for grade 2 hypertension (case 1); Another patient was found to have grade 1 hypertension and was not treated (case 2), and none of the patients had refractory hypertension. All patients denied any family history of cancer. One of the four cases had a family history of hypertension (case 2) (**Table 1**).

**Table 1:** Clinical features of four patients with adrenal CD.

	Case 1	Case 2	Case 3	Case 4
Sex/age	M/58	M/55	F/45	F/26
Complaint				
Accidental tumor	Yes	Yes	Yes	Yes
Fever	No	Yes	No	No
Joint pain	No	No	No	No
Headache	No	No	No	No
Sweat	No	No	No	No
Palpitations	No	No	Yes	No
Fatigue	No	No	No	No
Nocturia	No	No	No	No
Hypertension	Yes	Yes	No	No
Blood pressure, mmHg	164/88	144/76	120/80	110/70
Hypertension family history	No	Yes	No	No

### Laboratory investigations and endocrine parameters

Serum potassium, sodium, creatinine, and liver function were found to be almost normal in all four patients. One patient had increased urinary potassium excretion in 24 hours, but the serum potassium level was normal (case 2). The human immunodeficiency virus antibody was also negative in all patients. C-reactive protein was normal in cases 1 and 2. The diurnal cortisol rhythms of four patients were basically normal. 24-hour urine vanillin mandelic acid was normal in cases 3 and case 4. Blood dopamine, methoxyepinephrine, methoxynorepinephrine, epinephrine, norepinephrine, and methoxylamine were all in the normal range in case 1 and case 2. The Postural stimulation test showed that case 2 had elevated renin in supine and upright positions, but normal aldosterone levels. 24h urinary aldosterone was normal in all cases (**Table 2**).

**Table 2:** Laboratory investigations of four patients with adrenal CD.

	Case 1	Case 2	Case 3	Case 4
Clinical time (year)	2022	2018	2010	2008
Serum sodium, mmol/L	144.3	141	142.7	141.3
Serum potassium, mmol/L	3.64	4.21	3.78	3.9
Serum creatinine, umol/L	84.4	68.1	56.3	47
24 h urine potassium excretion, mmol/24h	29.2	65.3	23.6	18.3
ALT, U/L	11	13.7	13.3	9.7
LDH, U/L	162.1	142.7	115.6	106
WBC, 10^9/L	3.71	6.55	6.63	3.77
Neutrophils	0.723	0.529	0.755	0.721
CRP, mg/dl	<0.05	0.05	–	–
DHEA-SO4, (80-560ug/dl)	103	–	–	–
T, (male: 4.27-28.24nmol/L; female: 0.31-1.66nmol/L)	17.06	14.39	1.94	–
Plasma cortisol (0 am), (0-165.7nmol/l)	127.47	64.64	45.9	29.1
Plasma cortisol (8 am), (198.7-797.5nmol/l)	426.18	303.02	297.8	300.9
Plasma cortisol (4 pm), (85.3-459.6nmol/l)	283.72	181.58	165.6	185
ACTH (0 am), (<10.12pmol/l)	6.32	3.2	<2.2	<2.2
ACTH (8 am), (<10.12pmol/l)	13.7	5.97	4.3	3.9
ACTH (4 pm), (<10.12pmol/l)	10.4	5.71	<2.2	3.7
LDST (1mg) cortisol (8 am), nmol/L	46.99	–	–	–
24h Urinary Free Cortisol (98-500.1nmol/l)	324.8	72.9	–	–
24h Urinary VMA, umol/24h	–	–	22.3	25.5
DA (<196 pmol/l)	33	36.4	–	–
MN (≤420.9 pmol/l)	118.4	223.5	–	–
NMN (≤709.7 pmol/l)	173.8	512.5	–	–
E (≤605.9 pmol/l)	124.2	400.6	–	–
NE (413.9-4434.2 pmol/l)	466.7	578.9	–	–
3-MT (≤100 pmol/l)	72.3	42.6		
Postural stimulating test				
Supine PRA (2.8-39.9 uIU/ml)	<0.5	15.30	2.46	2.71
Supine aldosterone (3.0-23.6 ng/dl)	9.70	3.30	5.94	6.21
Upright PRA 2h (4.4-46.1 uIU/ml)	3.30	71.80	21.32	20.83
Upright aldosterone 2h (3.0-35.3 ng/dl)	3.70	6.20	13.39	12.97
24h Urinary aldosterone (1.19-28.1 ug/24h)	2.00	2.90	5.2	4.7

–, undone; GFR, glomerular filtration rate; ALT, aspartate transaminase; LDH, lactate dehydrogenase; WBC, white blood cells; CRP, C-reactive protein; DHEA-SO4, dehydroepiandrosterone; T, testosterone; ACTH, adrenocorticotropic hormone; LDST, Low-dose adrenocorticotropic hormone stimulation testing; VMA, vanillylmandelic acid; DA, dopamine; MN, Methoxyepinephrine; NMN, Methoxynorepinephrine; E, epinephrine; NE, norepinephrine; 3-MT, Methoxylamine; PRA, plasma renin activity.

### Imaging

Tumor size in the four patients determined by either CT or MRI ranged from 87 × 79 × 85 to 46 × 33 × 40 mm. In case 1, abdominal CT showed a solid-cystic mass with heterogeneous wall thickness in the right adrenal gland, and multiple mildly enlarged lymph nodes in the retroperitoneum. The enhanced scan showed marked enhancement of the wall and solid nodules. Positron emission tomography/computed tomography (PET-CT) suggested a cystic mass in the right adrenal gland with elevated metabolism in the solid portion, surrounded by scattered hypermetabolic lymph nodes. The maximum standardized uptake value (SUVmax) was 3.7. Imaging of the mass gave suspicion of malignancy or possibly pheochromocytoma. In case 2, enhanced CT showed a blood-rich mass of 46 × 33 × 40 mm in size with calcification and patchy unreinforced areas in the left adrenal gland with a CT attenuation value of 37.8 HU, which could indicate a pheochromocytoma.

**Figure 1:**
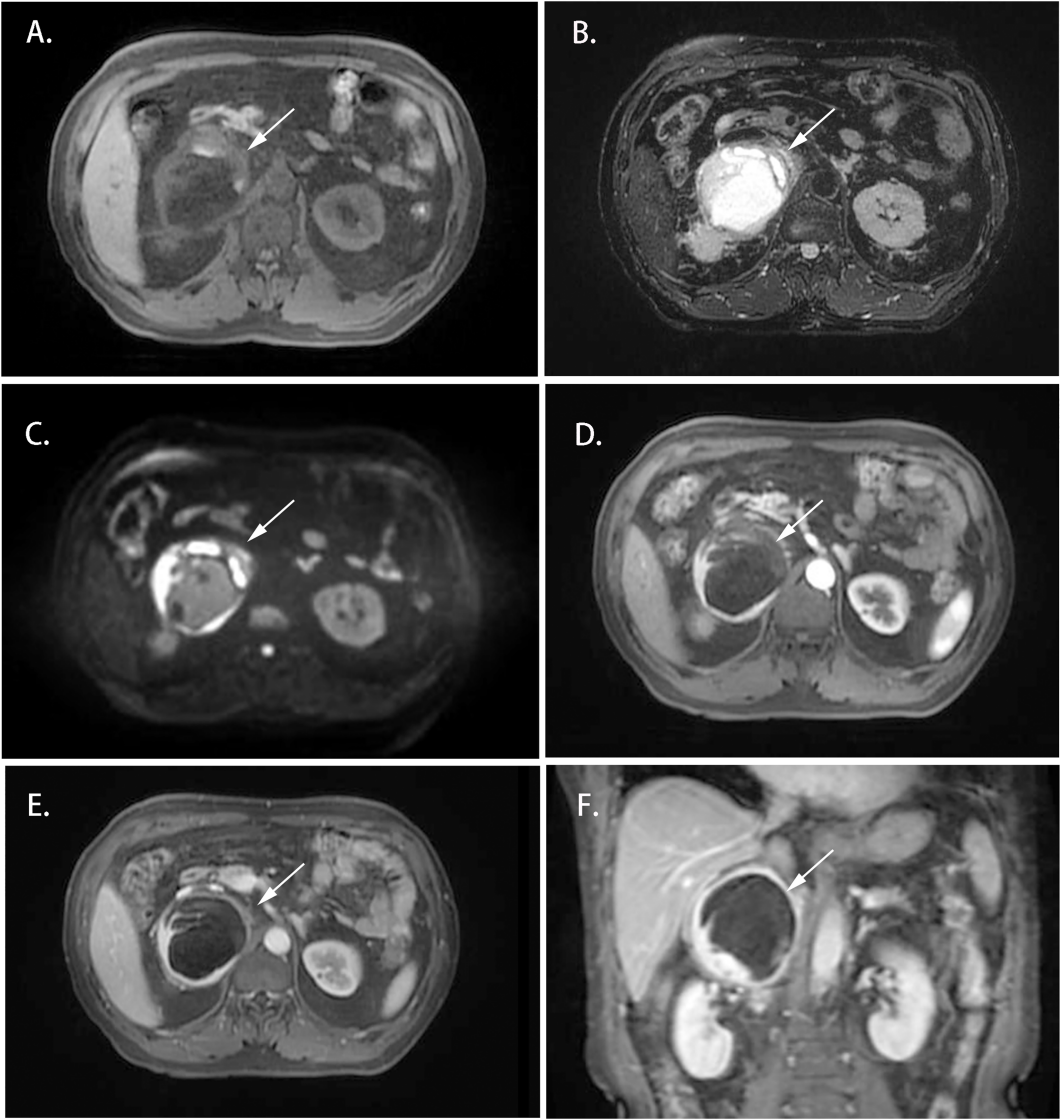
Magnetic resonance imaging of the Adrenal CD of Case 1 (white arrow). **(A)** Axial T1WI; **(B)** Axial T2WI; **(C)** DWI; **(D)** Enhanced T1WI (cortex phase); **(E)** Enhanced T1WI (delayed phase); **(F)** Coronal Enhanced T1WI. MRI scan and dynamic enhancement showed an 87 × 79 × 85 mm mass with partial hyper-intensity on T2WI and mild to moderate heterogeneous enhancement at the edge and septum of the lesion in the right adrenal region.

**Figure 2:**
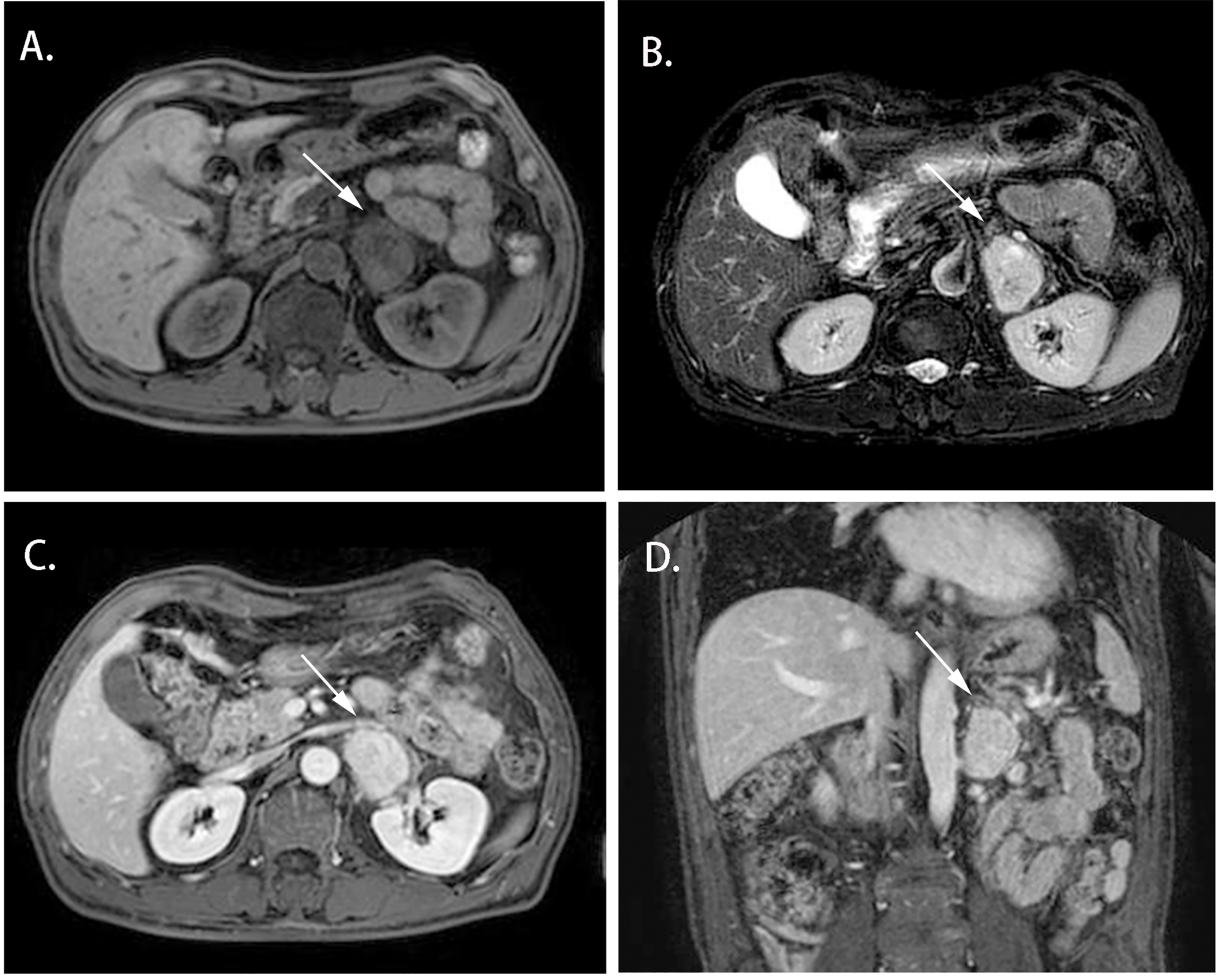
Magnetic resonance imaging of the Adrenal CD of Case 2 (white arrow). **(A)** Axial T1WI; **(B)** Axial T2WI; **(C)** Enhanced T1W1; **(D)** Coronal Enhanced T1W1. MRI scan and dynamic enhancement showed a 46× 33× 40 mm mass with hypo-intensity signal on T1WI and hyper-intensity on T2WI and with mild to moderate heterogeneous enhancement in the left adrenal region.

Cases 1-4 shared similar MRI images: tumor size larger than 4 cm^3^, relatively clear boundary, slightly long T1 signal, and heterogeneous long T2 signal (**Figures 1**, **2**). The lesion of case 1 manifested with a hypo-intensity signal on T1WI inside the tumor, and a hyper-intensity on T2WI characterized a “bulb sign” with the pseudocapsule. DWI images showed a hyper-intensity signal at the margin and internal septum of the lesion. Continuous mild to moderate enhancement of the margin and internal septum of the lesion was observed on the arterial and venous phases of T1WI, with no enhancement in the cystic center (**Figure 1**). Case 2-4 had similar features on MRI, represented with hypo-intensity signals on T1WI and hyper-intensity signals on T2WI. Enhanced images obtained in the arterial and venous phases showed moderate and uneven enhancement in mass (**Figure 2**). All the cases were considered to have imaging features of pheochromocytoma before surgery.

### Surgery and pathology

**Figure 3:**
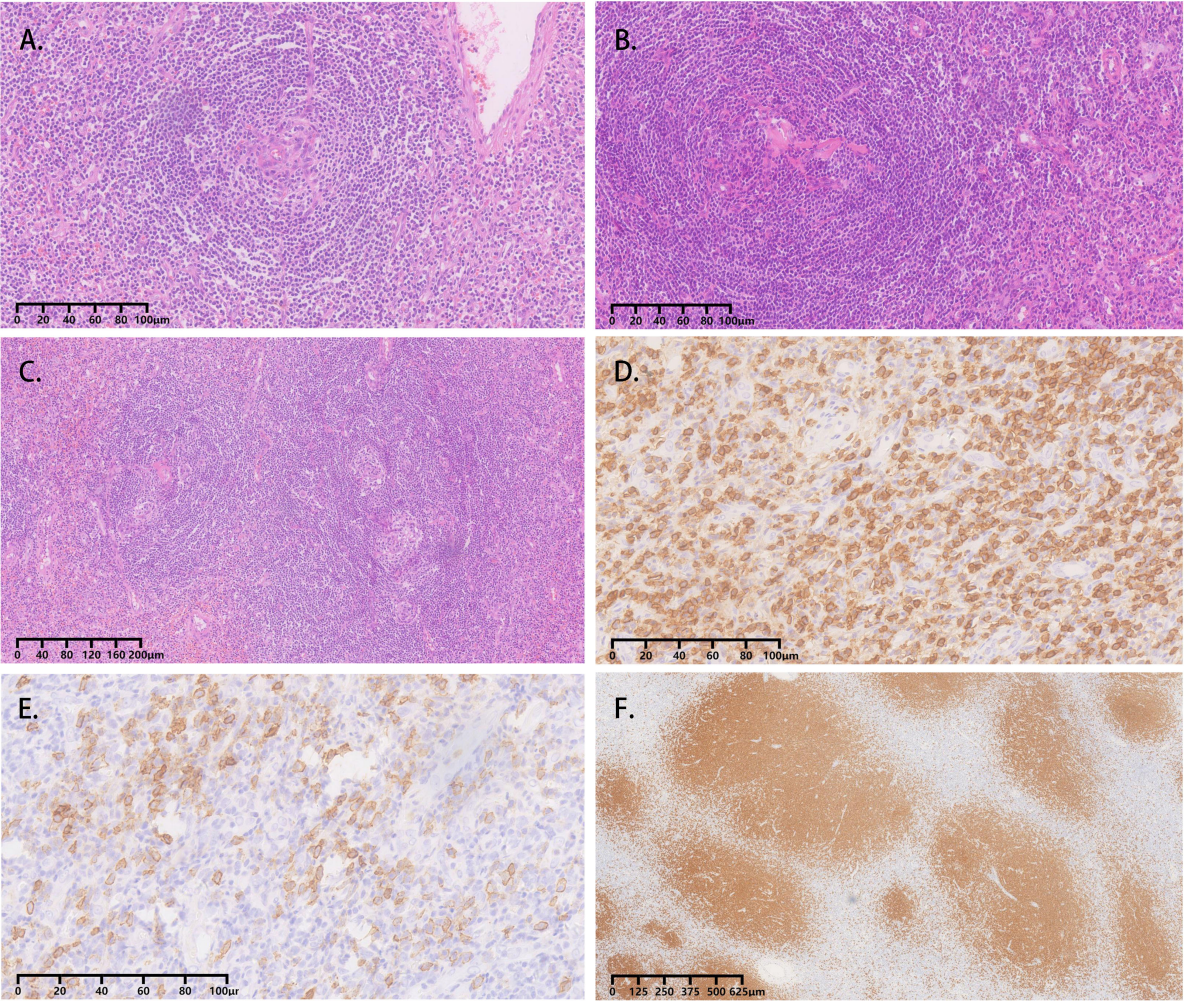
Adrenal CD, plasma cell variant histopathology in case 1. **(A-C)** Atretic germinal centers were traversed by sclerotic penetrating vessels and hyalinization, like lollipop follicles. Mantle zones were thickened with lymphocytes arranged in layers. Mantle zones may fuse and contain more than one germinal center, and follicular dendritic cells showed dysplastic features. Interfollicular areas and medulla contain sheets of small, mature plasma cells; **(D)** Clusters of plasma cells in the interfollicular areas were positive for CD138; **(E)** Immunohistochemical stain for CD38 identifies plasma cells; **(F)** The CD20 staining showed the expanded mantle zones.

All four cases underwent adrenal mass resection. They received α-receptor blockers according to preoperative preparations because pheochromocytoma/paraganglioma could not be ruled out. 2 cases were treated with the Da Vinci robot (cases 1, 2), while the operation was converted to laparotomy because of the large tumor volume and severe adhesion in case 1, and the other 2 cases had laparoscopic surgery (cases 3, 4). All operations were successful without complications. All cases were intra-adrenal. The tumors were all well-circumscribed, firm, and grayish-white to yellowish-brown in color (**Table 3**). The hematoxylin and eosin (H&E) stain of case 1 presented with adrenal lymphoid hyperplasia, and atretic germinal centers were traversed by sclerotic penetrating vessels and hyalinization, like lollipop follicles. Mantle zones were thickened with lymphocytes arranged in layers. Mantle zones may fuse and contain more than 1 germinal center, and follicular dendritic cells show dysplastic features. Interfollicular areas and medulla contain sheets of small, mature plasma cells, and it showed histological features of a plasma cell type (**Figures 3A–C**). Immunohistochemical staining revealed that CD138 (plasma cell), CD38 (plasma cell), CD20 (B cell) (**Figures 3D–F**), CD3 (T cell), Bcl-2, and Ki-67 (germinal centers +90%) of case 1 were positive. No metastases from three retroperitoneal lymph nodes were detected by freezing during the operation. In cases 2-4, histopathology revealed lymphoid follicles with atrophic germinal centers, multiple germinal centers within the same mantle zone, an “onion skin” appearance at the periphery of the follicular, and increased vascularity with hyalinization. Immunohistochemical staining revealed that CD31 and CD34 were positive in cases 2–4. Pathology indicated that case 1 was consistent with plasma cell type, and the other 3 cases were diagnosed as hyaline vascular type (cases 2-4).

**Table 3:** Surgery and pathology of four patients with adrenal CD.

	Case 1	Case 2	Case 3	Case 4
Surgery	Adrenalectomy	adrenal masses resection	adrenal masses resection	adrenal masses resection
Surgical method	Laparotomy	Da Vinci robot	Laparoscopic	Laparoscopic
Preoperation diagnosis	Pheochromocytoma	Pheochromocytoma	Pheochromocytoma	Pheochromocytoma
Preoperative treatment	Phenoxybenzamine	Phenoxybenzamine	Phenoxybenzamine	Phenoxybenzamine
Postoperation Treatment	No	No	No	No
Blood pressure (mm Hg)	141/71	103/62	123/78	110/70
Pathological Type	Plasma cell	Hyaline	Hyaline	Hyaline
Location (adrenal gland)	Right	Left	Right	Left
Size (mm)	100x80x50	60x50x30	60x45x40	75x60x50
Character	Moderate	Moderate	Soft	Moderate
Plane color	Gray&red	Gray&red	Gray	Faint yellow
Lymph node	Right retroperitoneal	No	No	Around
Amount	3	–	–	7
The biggest (mm)	45x25x10	–	–	15x10x10

adrenal masses resection refers to adrenal-preserving surgery of adrenal tumours. The “-” means that case 2 and 3 have no lymph nodes around adrenal masses, that is, the “amount” of lymph nodes is 0 without “the biggest size”.

### Follow-up

All four cases are alive without relapse after a median follow-up of 8 years, and no chemotherapy or steroid replacement therapy was administered after the operation.

## Discussion

Over the past few decades, there has been a significant increase in the use of diagnostic imaging techniques, resulting in a subsequent rise in the incidental discovery of adrenal masses. Nonfunctional adrenocortical adenomas are the most common adrenal incidentalomas, followed by hormone-producing adenomas, and adrenocortical carcinomas (11–13). Adrenal CD is relatively rare, and the features of adrenal CD are less well described. Our case series report illustrated the baseline characteristics and outcomes of adrenal UCD from the Chinese PLA General Hospital, a type of adrenal incidentaloma lacking specific clinical and biochemical features. These patients underwent adrenal mass resection, and indicated a favorable prognosis.

The CD was first described by Benjamin Castleman in 1954 in a group of patients with mediastinal lymph node enlargement (14). Epidemiological studies show that the prevalence of CD is about 21-25 cases per million people, with a median age of onset of 34–40years (6). UCD can involve many sites, commonly in the neck, abdomen, and mediastinum (8, 15). Retroperitoneum is also a common site of UCD, and the number of the adrenal UCD lesions was found to be about 12% in retroperitoneal UCD (7). Patients with adrenal UCD ranged in age from 16 to 70 years with an equal sex ratio and localization (**Table 4**). Patients with UCD usually have no obvious clinical symptoms or abdominal discomfort, and their conventional biochemical and adrenal function examinations do not show obvious abnormalities (16–25). In other reports, the patients were accompanied by hypertension, weight loss, paraneoplastic pemphigus, and pheochromocytoma (18, 26–30). In a rare case report, hyperandrogenism was found in a female patient with adrenal UCD, and the author attributed this phenomenon to enhanced 5α-reductase activity associated with IL-6 (28). However, adrenal UCD usually has a relatively large volume, which is different from a nonfunctional adrenocortical adenoma (33). In the present study, cases 1–4 had adrenal incidentalomas found on imaging with no local or systemic symptoms.

**Table 4:** Reported cases of adrenal Castleman’s disease.

Author	Sex/age	Presenting symptom	Location (adrenal gland)	size(mm)	CT finding	Calcification	Necrosis	Pathological Type	Prognosis
Maghsoudi et al. (16)	F/29	abdominal pain	right	50 × 45	No contrast enhancement	No	No	HV	NA
Raja et al. (17)	M/16 (26)	left upper abdomen pain, weight loss	left	53 × 51 × 55	Enhancing heterogenous mass	No	Yes	HV	No recurrence at 6 months
Sundar et al. (18)	F/44	abdominal discomfort	right	55 × 33 × 67	Enhancing heterogenous mass	No	No	HV	No recurrence at 6 months
Gupta et al. (19)	M/25	abdominal discomfort	right	60 × 80	NA	Yes	No	HV	NA
Santomauro et al. (20)	M/16	incidentaloma	left	48	Enhancing mass	No	No	HV	NA
Otto et al. (21)	M/33	incidentaloma	right	~40-50	small foci of enhanced density	No	No	HV	NA
Cao et al. (22)	F/51	abdominal pain	left	90 × 80	NA	No	No	HV	No recurrence at 4 years
Cao et al. (22)	M/56	incidentaloma	left	80 × 58	NA	No	No	HV	No recurrence at 3 years
Hong et al. (23)	F/65	abdominal discomfort, hypertension	left	50	Enhancing mass with peripheral enhancement, retroperitoneal lymph nodes	No	No	HV	NA
Guo et al. (24)	M/59	abdominal pain	left	46 × 51	Enhancing heterogenous mass	No	No	HV	NA
Romero et al. (25)	F/70	abdominal fullness	left	72 × 40 × 38	Enhancing mass with retrocrural, abdominal aortic, and inguinal lymph nodes	Yes	No	PC	NA
Mussig et al. (2007) (26)	M/51	hypertension, overweight	right	30	Enhancing homogenous mass, retroperitoneal lymph node enlargement	No	No	HV	No recurrence at short-term
Zhang et al. (27)	F/62	hypertension, low white blood cell count	right	65	Enhancing mass with multiple hypodensities of varying sizes	Yes	No	HV	No recurrence
Mussig et al. (2006) (28)	F/32	hypertension, hyperandrogenism	left	16 × 42 × 9	Enhancing homogenous mass	No	No	HV	No recurrence at 4 years
Shi et al. (29)	M/31	Paraneoplastic Pemphigus	right	10 × 9 × 8	Enhancing mass	No	No	HV	No recurrence at 4 years
Stelfox et al. (30)	M/44	type I neurofibromatosis, mitral valve endocarditis, Pheochromocytoma	left	43 × 30 × 18	Right adrenal and left retroperitoneal abdominal masses	No	No	HV	NA
Chen et al. (31)	F/26	left flank pain	left	40 × 30	Enhancing heterogenous mass	No	No	NA	No recurrence at 12 months
Chen et al. (32)	F/51	poor appetite, weight loss	left	52 × 47 × 56	Enhancing heterogenous mass	No	Yes	HV	No recurrence at 24 months

HV, hyaline vascular; PC, plasma cell.

Large adrenal gland mass usually requires a systematic evaluation of adrenal endocrine workup to assist in diagnosing the mass from the perspective of endocrinology diagnosis and treatment (11). Our data showed that two patients had elevated midnight cortisol and one patient had mildly elevated ACTH, but no obvious abnormalities in aldosterone or catecholamine metabolism was found. These biochemical abnormalities are not representative, and we did not see significant adrenal insufficiency, or excess production of adrenal hormones either, even in the case of a larger adrenal CD. This phenomenon is plausible, given that the cases were all unilateral non-functional adrenal lymphocytic hyperplasia. It is worth noting that hypertension may be present in some patients with adrenal UCD (23, 26–28). This may be attributed to the differential diagnosis of adrenal mass, since adrenal mass is a major cause of secondary hypertension.

Imaging findings of adrenal UCD are related to discriminating features and also with the pathological types. Hyaline vascular adrenal CD usually appears as a soft tissue mass with uniform density and well-defined borders on CT and with uneven density in a few lesions (16–18, 20, 21, 23, 24, 26–32). The plasma cell type adrenal UCD was rare, and had only been reported in 1 previous case, according to our review of the literature (25). Despite the controversy, the plasma cell type UCD is less common, larger in size, and more likely to have necrosis, peripheral enhancement, and the presence of enlarged retroperitoneal lymph nodes than the hyaline vascular type, contributing to malignant morphological features (25, 34–36). In our report, the imaging results in plasma cell type adrenal UCD in case 1 showed a large mass with apparent necrosis and peripheral enhancement. Cases 2-4 were hyaline vascular type adrenal UCD; their mass bodies were relatively smaller and the mass density was relatively uniform, compared with case 1.

As a less common disease, adrenal UCD is easily misdiagnosed. Physicians often need to differentiate adrenal CD from pheochromocytoma, adrenal lymphoma, and cortical carcinoma. Pheochromocytoma patients usually present with sweating, headache, tachycardia, and hypertension (37). However, the presence of silent pheochromocytoma or pheochromocytoma with less typical signs should also be considered, which may also cause adverse cardiovascular events, as well as severe intraoperative blood pressure fluctuations (38). Pheochromocytoma with a rich blood supply presents with a hyper-intensity signal on T2WI, strong enhancement after contrast agent administration, and liquefaction necrosis frequently occurring. Adrenal CD, especially plasma cell variant adrenal UCD, manifested with an isohypointense T1W1 signal and a characteristically hyperintense T2W1 signal and is sometimes accompanied by necrosis or cystification in larger adrenal CD, making it more difficult to distinguish from pheochromocytoma and adrenal cortical carcinoma. Hyperandrogenism and cortisol autonomous hypersecretion with tumor metastasis often occur in adrenocortical carcinoma, which can help distinguish it from adrenal CD. Fever with lymphadenopathy makes it difficult to differentiate adrenal CD from adrenal lymphoma, while the latter often presents with an adrenal mass with uniform density involving bilateral adrenal glands.

PET-CT has a certain diagnostic value in differentiating pheochromocytoma from UCD. A study by Jiang et al. reported the major points of ^18^F fluorodeoxyglucose (FDG) PET-CT to differentiate paragangliomas from UCD. Paragangliomas usually have higher SUV values, and the SUVmax > 7.75 may be used as a cut-off point to differentiate paragangliomas from UCD. In this study, the SUVmax of adrenal UCD in case 1 was 3.7, which is consistent with Jiang’s findings (9). Plasma free metanephrines or urine fractionated metanephrines also have certain differential diagnosis values, but there are still some pheochromocytomas or paragangliomas that cannot be distinguished by biochemical detection (39). All cases in this report were misdiagnosed as pheochromocytomas with preoperative imaging, and phenoxybenzamine was used for preoperative preparation in all 4 cases. There are a few report of patients with both pheochromocytoma and UCD (30, 40). Therefore, it is prudent to apply alpha-blockers preoperatively to avoid severe intraoperative complications with the omission of pheochromocytoma (9).

A definitive diagnosis of adrenal CD or lymphoma is important and challenging because the preferred therapies for lymphoma are usually not surgical, and CD may be a pre-disease form of lymphoma (35, 41). Adrenal lymphomas are usually large and well-defined masses, and some have bilateral involvement of the adrenal glands. Adrenal lymphomas have homogeneous enhancement and are less likely to have calcification on CT imaging. On a CT scan, the hyaline vascular type of adrenal UCD appears more similar to adrenal lymphoma than the plasma cell type of adrenal UCD. However, it is possible to differentiate between hyaline vascular type adrenal UCD and lymphoma by observing the presence of calcification and heterogeneous enhancement in the former (19, 25, 27, 35). In our case series, one case (case 2) was found to have a blood-rich occupancy with calcification, but the high SUVmax values in PET-CT raised the suspicion of lymphoma. Patients with adrenal lymphoma often present with systemic symptoms, including back pain, fever, night sweats, and weight loss. Bilateral adrenal involvement may also lead to adrenal insufficiency in affected individuals. Higher LDH and β2-microglobulin levels in the biochemistry tests imply the diagnosis of adrenal lymphoma; in contrast, the adrenal CD is usually unilateral with no definitive biochemical abnormalities (42, 43). An adrenal puncture biopsy can be considered in cases where a definitive diagnosis cannot be made based on imaging and clinical features (25, 44). Further accumulation of clinical and imaging features of plasma cell type adrenal UCD is required.

Surgical resection is the primary treatment option for UCD (8), and the prognosis is usually prospective if the lesion can be completely removed irrespective of the pathology, clinical presentation, and highly expressed Ki67 index (17, 18, 22, 26–29, 31, 32, 45). Anti-IL-6 antibody therapy or radiotherapy can be considered for those unresectable UCD masses because of their size or location (46). Talat et al. summarized 278 cases of UCD and found that the 3-year and 5-year postoperative progression-free survival rates were 89.7% and 81.2%, respectively, and the 10-year overall survival rate was 95.3% (8). Similar results also been reported in research by Zhang et al. (47). However, an increased risk of paraneoplastic pemphigus, bronchiolitis obliterans, AA amyloidosis, and lymphoma has been reported in CD patients (35, 48, 49), suggesting that adrenal CD patients with persisting constitutional symptoms still need further follow-up despite complete excision.

## Conclusions

Adrenal Castleman disease is a proliferative disease of the lymphoid tissue occurred in the adrenal region and is difficult to distinguish from pheochromocytoma and lymphoma on imaging. MRI, ^18^F PET-CT, and adrenal function assessment are helpful in the diagnosis of adrenal UCD. Surgery is the mainstay of treatment, and the prognosis is usually promising.

## Data availability statement

The original contributions presented in the study are included in the article/supplementary material. Further inquiries can be directed to the corresponding authors.

## Ethics statement

The studies involving human participants were reviewed and approved by the Ethics Committee of the First Medical Center of Chinese PLA General Hospital. The patients provided their written informed consent to participate in this study.

## Author contributions

Conceptualization and writing: HY, YW, and LZ. Review and editing: YM, and LZ. Instrumental in revising the manuscript: YL, JD, QG, WG, ZL, and JTD. All authors contributed to the article and approved the submitted version.
